# Recreational substance use is linked with difficulty in recalling personal experiences

**DOI:** 10.1038/s41598-025-13800-y

**Published:** 2025-10-03

**Authors:** Adnan Levent, Eddy J. Davelaar

**Affiliations:** 1 Department of Psychology, Royal Holloway, University of London, Egham Hill, Egham, TW20 0EX UK; 2 School of Psychological Sciences, Birkbeck, University of London, London, UK

**Keywords:** Recreational substance use, Autobiographical memory, Episodic memory, Cannabis use, Cocaine use, Ecstasy use, Cognition, Neuropsychopharmacology, Psychology, Human behaviour

## Abstract

Recreational use of substances such as cannabis, MDMA and cocaine is thought to harm the neurotransmitter communication networks that coordinate many memory processes that support autobiographical memory (AM). Research on the impact of substance use on AM is limited and primarily focused on cannabis use or individuals with substance dependence. Additionally, previous studies mainly examined broad AM characteristics (e.g., specific vs. non-specific memories) without exploring the specific characteristics of recalled memories. In the present study, the possible consequences of recreational substance use on AM were investigated to provide a better understanding of the specific aspects of AM that are most vulnerable to substance use. The study included 100 participants aged 18–55, consisting of 47 individuals who did not use substances and 53 individuals who reported substance use. All participants completed self-report questionnaires and participated in a lab-based autobiographical memory test. The results revealed that participants who reported recreational substance use recalled significantly fewer specific personal event memories than participants who did not use substances and were also more likely to omit a response within the time limit. The results remained significant after controlling for covariates, such as general health, sleep routine, alcohol use and age. This research contributes to the body of knowledge on substance-related impairments in AM and suggests that even occasional recreational substance use may impair specific AM retrieval.

## Introduction

Despite the risks, recreational substance use (the term used to describe non-dependent casual substance use) remains high worldwide. Although alcohol, tobacco, and caffeine are also classified as substances, this study focused on substances such as cannabis, MDMA, and cocaine. Distribution, possession, and the recreational use of these substances is illegal in the United Kingdom where the current study was conducted. According to the United Nations Office on Drugs and Crime’s World Drug Report 2022, 284 million people aged 15–64 used substances at least once in the previous year, up from 226 million in 2010 (26% increase). This is around 1 in every 18 persons, or 5.6% of the population of the world in that age group. The Report further noted that substance use is much more common among young people^1^.

While the exact mechanisms through which recreational substances such as cannabis, MDMA and cocaine affect cognition remain not fully understood, several theoretical frameworks and empirical findings offer valuable insights. These substances appear to influence memory by interacting with neurobiological systems that underpin core cognitive processes, such as learning, attention, and memory encoding and retrieval. For example, cannabis is thought to impact cognition through its interaction with the endocannabinoid system (ECS), which comprises endogenous cannabinoids, cannabinoid receptors (CBs), and regulatory enzymes^2^. Δ9-tetrahydrocannabinol (THC), the primary psychoactive component of cannabis, binds to CBs^3,4^, which are highly concentrated in brain regions critical for memory function, namely the hippocampus, amygdala, basal ganglia, and prefrontal cortex^5,6^. Disruption to this system is thought to impair neurobehavioral processes, including learning and memory^7–10^.

MDMA primarily influences neurotransmission by promoting the release of serotonin (5-HT), a neurotransmitter that plays a critical role in memory^11,12^. This suggests that MDMA’s cognitive effects may be largely mediated by its impact on the serotonergic system. Indeed, the well-established link between long-term MDMA use and reduced serotonin signalling is supported by neuroimaging studies demonstrating decreased serotonin transporter binding in the frontal, parietal, and temporal lobes^13–17^.

Cognitive deficits associated with cocaine, which primarily interacts with the dopamine neurotransmitter system, are believed to result from disruptions within this system. Dopamine plays a crucial role in memory, executive functions, and attention. For example, dopamine neurons increase their firing in response to salient stimuli in brain regions such as the hippocampus and ventral tegmental area, both involved in encoding new episodic memories^18^. Additionally, dopamine receptors (D1 and D5) enhance glutamatergic transmission, facilitating memory encoding^19^. Cocaine has been shown to reduce dopamine neuron activity, leading to decreased dopamine release and receptor function^20^ which likely underlies its cognitive effects.

As a consequence, recreational use of those substances use has been consistently linked to a range of cognitive impairments, such as prospective memory impairments^21,22^, reduced verbal learning^23–26^, and executive dysfunctions^27–30^.

An important consideration when interpreting the cognitive effects of these substances is the high prevalence of polysubstance use**.** Many individuals who report using one substance also engage in the use of others which introduces complexity when trying to isolate the impact of a single substance on cognitive outcomes. For example, Grov et al.^31^ assessed 400 individuals with a history of substance use aged 18–29-year-old between 2004 and 2006 and found that 91.7 per cent of the participants had engaged in polysubstance use. Most of them tended to combine ecstasy and cocaine, making it difficult to attribute observed cognitive deficits to one substance alone.

Autobiographical memory (AM) refers to memory for one’s personal history which is thought to be a vital part of one’s life since it aids in self-awareness, interpersonal connections, decision-making, and stress management^32^. There is very limited research on the effects of recreational substance use on AM. Existing studies predominantly focus on the effects of cannabis, suggesting that it is associated with AM impairments, particularly in people with heavy substance use^33,34^. A study on individuals with substance dependence also reported AM deficits^35^.

A further limitation of the existing literature is the generalised approach to assess AM, with studies often focusing on broad AM characteristics, such as specificity (specific vs. non-specific memories) without exploring the detailed features of recalled memories. For example, Mercuri et al.^33^ only evaluated whether memories were specific versus non-specific, while Pillersdorf and Scoboria^34^ examined the proportion of over-general memory responses among individuals with and without substance use history and Oliveira et al.^35^ assessed whether participants recall the requested information or not in the given time. A more in-depth analysis of recalled memory characteristics is essential to identify distinct memory processes affected by substance use, providing a better understanding of the aspects of AM that are most vulnerable to impairment.

Therefore, the present study aimed to fill this gap by examining the detailed effects of recreational use of substance such as cannabis, MDMA and cocaine use on AM, offering insights into the cognitive mechanisms affected by substance use. Following recommendations for substance-use studies outlined in a systematic review by Levent and Davelaar^21^, 100 participants aged 18–55 were recruited from the general population. Participants who reported substance use were required to abstain from substance use for at least 7 days prior to participation. A comprehensive range of possible moderating variables, including age, ethnicity, fluid intelligence, sleep quality, general health, level of education, alcohol, and nicotine use, were examined and included as covariates in the statistical analyses due to their potential impact on cognitive functions^36–41^. It was hypothesised that recreational substance use would negatively impact AM.

## Methods

### Participants

The study included 100 participants from the general public: 47 individuals who did not use substances (27 females, aged 18–55) and 53 individuals who reported substance use (21 females, aged 18–55). Participants were recruited through a variety of methods, including advertising, social media, and word-of-mouth. Participants who reported past or current recreational substance use were assigned to the substance use group. There were no exclusions based on specific types of substances. All participants were residing in London and were either native speakers of English or were very fluent English speakers. All participants were required to abstain from substance use such as cannabis, MDMA and cocaine for at least 7 days and alcohol for at least 24 h before the study session. On the day of the experiment, participants verified their abstinence from recreational substance for a minimum of 7 days and alcohol for at least 24 h. However, no tests could be conducted to verify their abstinence. The pre-study questionnaire indicated no prior history of neurological or psychiatric conditions among participants, including neurodivergence or addiction. The study was approved by the Ethics Committee of Birkbeck University and was administered under the ethical guidelines of the British Psychological Society.

### Design and analysis

This study used a quasi-experimental design to compare the performance of individuals who reported recreational substance use with those who did not, on an autobiographical memory test. The independent variable was recreational substance use status and the dependent variable was performance on the autobiographical memory test. The datasets generated and analysed during the current study are available in the Open Science Framework repository, [10.17605/OSF.IO/VUJ3T]. The data were analysed using SPSS. Because most of the data were not normally distributed, the Mann Whitney U test was used instead of the independent t-test. When significant results were obtained, Quade’s rank analysis of covariance (RANCOVA) was applied to account for covariates, which has demonstrated its robustness and effectiveness, particularly in cases where data exhibits non-normal distributions^42,43^. To examine the relationships between the various variables, Spearman’s Rank-Order Correlation was utilised. To manage family-wise error rates, Holm-Bonferroni corrections were implemented.

### Materials

Background questionnaires were used to collect information about the sample population’s characteristics (e.g., gender, age, education level) and their current use of nicotine, alcohol, and other recreational substances.

*The 12 items short form of Raven’s Advanced Progressive Matrices* was used to measure participants’ fluid intelligence which consists of 12 incomplete 3 × 3 matrices, and participants must choose one of eight options to complete each matrix by following the rules within each row and column^44^.

*The General Health Questionnaire (GHQ)*^45^ was utilised to detect minor psychiatric disorders within the study’s sample population. The GHQ is a 12-item questionnaire that asks participants to rate their current state of mental health compared to their usual state. Each item is scored from 0 (not at all) to 3 (much more than usual). The total possible score ranges from 0 to 36, with higher scores indicating greater health-related concerns. The GHQ has been shown to be a reliable and valid tool for measuring psychological distress^46,47^.

*The Pittsburgh Sleep Quality Index* (PSQI)^48^ was employed to examine sleep quality experienced over the past month. It assesses seven domains, including sleep latency, sleep disturbances, and sleep duration. Each component is scored on a 0–3 scale, and the total score ranges from 0 to 21, with lower scores indicating better sleep quality. Previous studies have demonstrated that the PSQI exhibits good internal consistency, test–retest reliability, and validity^49,50^.

*The Autobiographical Memory Test (AMT)*^51^ was used to assess the ability to retrieve specific memories from autobiographical memory. Participants were told 5 negative (e.g., clumsy, angry, hurt, lonely, and sorry) and 5 positive cue words (e.g., happy, successful, surprised, safe, and interested) in random order one by one and given 30 s to generate a specific memory that happened on a particular day at least three months ago (to avoid the recency effect) and briefly describe this memory in response to each cue word. For practice, participants were presented with three neutral words (car, forest, and chair) and asked to provide a specific memory for at least two of the three words before completing the rest of the test. Participants’ responses were recorded and scored according to the criteria defined by Mark et al.^52^ as specific or non-specific memories. Specific memories were defined as events that occurred at a particular place and time within the course of one day (e.g., “I broke my arm while I was playing football on 22nd June 2015”). Non-specific memories included extended memories (events that lasted for a longer period of time; e.g., “I enjoyed my weekend in Berlin”), categoric memories (events that happened repeatedly over a period of time; e.g., “Whenever I go for a bike ride”), and non-memories (semantic associated; e.g., “I am a clumsy person”). The following subtests were reported: specific memory recall, non-specific memory recall (including extended memory recall, categorical memory recall and non-memory recall), and omission/no response for cases with no response (e.g., “I don’t know”) within the time limit. Subtest scores were calculated by counting the frequency of each memory type recalled per cue word, with a maximum score of 10 for each subtest. Additionally, an overall score was calculated by assigning points to each response based on its specificity: 4 points for specific memories, 3 for extended memories, 2 for categorical memories, 1 for non-memories, and 0 for omissions. These scores were then summed. The total score ranged from 0 to 40. A higher scores indicate better performance.

### Procedure

The study was conducted in two parts. In the first part, participants were sent a web link to an online survey which included self-report questionnaires, such as the GHQ and PSQI, ideally to be completed either on the same day as the testing session or up to one day prior. In the second part, participants were invited to attend the testing session, where they actively took part in the AMT. Participants were informed about the overall purpose of the experiment and provided written consent. After completing the study, participants received a detailed explanation of the experiment, an Amazon voucher, and educational materials on substance use awareness. This study is part of a comprehensive research project aimed at exploring the potential effects of recreational substance use on various cognitive abilities. Previously, we have published a detailed analysis addressing prospective memory (see Levent & Davelaar)^22^.

## Results

The demographic information of participants with and without a history of recreational substance use, along with data on alcohol and nicotine use, fluid intelligence, and health-related variables are presented in Table 1.

**Table 1:** Demographic information of participants with and without a history of recreational substance use together with alcohol/nicotine use, fluid intelligence, and health variables.

		Participants with substance use	Participants without substance use	Total
N		53	47	100
Gender (M/F)		32/21	20/27	52/48
Age	18–25	5	4	9
	26–30	9	6	15
	31–35	11	15	26
	36–40	11	7	18
	41–45	11	7	18
	46–50	3	7	10
	51–55	3	1	4
Ethnicity^a^	White	38	28	66
	Asian	4	11	15
	Black	5	5	10
	Mixed	3	3	6
	Other	3	0	3
Education level	Secondary	2	3	5
	College	7	8	15
	Bachelor	26	19	45
	Masters	14	15	29
	Advanced/PhD	4	2	6
Alcohol use***	Never	2	14	16
	Monthly or less	9	13	22
	2–4/month	17	15	32
	2–3/Week	18	4	22
	> 4/week	7	1	8
Nicotine use**	Never	28	42	70
	Several/month	8	2	10
	Several/week	6	2	8
	Once/day	4	0	4
	Several/day	7	1	8
RAPM	Median	10	11	11
GHQ**	Median	12	8	10
PSQI***	Median	6	3	5

** *p* < 0.01, *** *p* < 0.001.

^a^ The following classification of ethnicity was used: Asian includes British-Asian, Black includes Black-British, African, and Caribbean. RAPM, Raven’s advanced progressive matrices; GHQ, General health questionnaire; PSQI, Pittsburgh sleep quality index.

Chi-square tests were employed to examine the association between background variables and substance usage. Some of the cells in the table were combined for the Chi-square test because they had an expected count less than 5. For instance, in the case of alcohol use, the categories “never” and “monthly or less” were combined, as were “2 to 3 times a week” and “4 or more times a week”. Regarding age, 18–25 and 26–30 (e.g., 18–30), 31–35 and 36–40 (e.g., 31–40), 41–45, 46–50 and 51–55 (e.g., 41–55) were combined. As for educational level, “secondary and college degrees” were combined, as were “masters and advanced/PhD degrees”. In terms of ethnicity, participants were categorised as “white” and “non-white,” and for nicotine use, they were classified as “smoker” or “non-smoker.” The findings indicated that there were no significant distinctions between the two groups in terms of age, gender, educational attainment, or ethnicity. The majority of participants in both groups fell within the 26–45 years age range, identified as having a white ethnic background, and held either a BSc or MSc degree. However, there was a group difference with respect to nicotine and alcohol use: individuals who reported recreational substance use included a higher proportion of smokers (*χ*^*2*^(1, N = 100) = 15.83, *p* < 0.01) and more frequent alcohol consumption (*χ*^*2*^(2, N = 100) = 19.91, *p* < 0.001) than individuals without a history of substance use. Nicotine use was related to cannabis use, with 35.7% of non-smokers and 76.7% of smokers using cannabis (*χ*^*2*^(1, N = 100) = 14.11*, p* < 0.001). Mann–Whitney U-tests showed that individuals who reported substance use had significantly more problems with sleep (*U* = 644.5, *p* < 0.001) and general health (*U* = 850.5, *p* < 0.01) compared with individuals who did not report substance use. There was no group difference on intelligence (*p* > 0.81). As nicotine use was specifically associated with cannabis use, only alcohol consumption, GHQ and PSQI scores were used as covariates in further analyses. Additionally, due to the age span within the sample, age was incorporated as a covariate in further analyses, given prior research suggesting that ageing can have an adverse impact on autobiographical memory^38,39^.

There were no correlations between AM and the general health and sleep quality covariates, apart from a few weak correlations. However, those correlations did not remain significant after Holm-Bonferroni correction, thus they were not reported. One way ANOVAs were run to assess the relationship between AM performance and the other covariates (e.g., age and alcohol use). Only alcohol use was associated with autobiographical memory as 2 to 3 times a week alcohol users (*M* = 5, *SD* = 1.98) scored worse than non-alcohol users (*M* = 7.31, *SD* = 2.7) in the AMT specific subtest (*F* (4, 95) = 3.56, *p* = 0.009). A one-way ANCOVA was conducted to determine significant differences between alcohol user groups on AM while controlling substance use status (users and non-users). The results failed to remain significant (*F* (4, 94) = 0.941, *p* = 0.444), suggesting that substance use may moderate the association between alcohol use and AM. Furthermore, there were no associations between age and AM. Nevertheless, alcohol use and age were included as covariates, along with general health and sleep quality in further analyses.

As seen in Table 2, the majority of participants with a history of substance use (72%) reported using more than one type of substance (i.e., polysubstance use). They typically consumed cannabis, MDMA/ecstasy and cocaine. Data on the dosage of these substances used was not collected. See Figure S1 in Supplementary Material for an overview of substance use profiles and the number of participants for each profile. The majority of them use substances rarely (1 or 2 times every three months) or occasionally (1 or 2 times a month). There were also participants who previously used certain substances, primarily cannabis. However, it should be noted that some of these individuals currently reported using other substances. For example, five participants who no longer used cannabis reported current use of cocaine, and four reported current use of ecstasy.

**Table 2:** Frequency of substance use among participants reporting use.

	1	2	3	4	5	6	Total
Cannabis	14	16	10	3	2	3	48
Cocaine	4	9	11	10	2	1	37
MDMA or ecstasy	6	11	13	6	0	0	36
GHB	3	9	5	1	0	0	18
Hallucinogenic	4	11	1	0	0	0	16
Ketamine	7	9	1	0	0	0	17
Methamphetamine	6	4	1	1	0	0	12
Mephedrone	4	4	2	1	0	0	11

1 = Ex-users; 2 = Very Rarely: 1 or 2 times a year; 3 = Rarely: 1 or 2 times every three months; 4 = Occasionally: 1 or 2 times a month; 5 = Frequently: 1 or 2 times a week; 6 = Very Frequently: 3 or more times a week.

Table 3 shows the results. Participants with a history of recreational substance use recalled significantly fewer specific autobiographical memories (*U* = 492.5, *p* < 0.001), more nonspecific memories (*U* = 783, *p* < 0.001), and had more omissions (*U* = 581.5, *p* < 0.001) than participants without a history of recreational substance use. The results remained significant after statistically controlling for the covariates (e.g., sleep quality, general health, alcohol use and age), but the effect on non-specific memories did not survive the Holm-Bonferroni correction.

**Table 3:** Mann–Whitney and RANCOVA tests’ results for the AMT.

	Participants with substance use (53)		Participants without substance use (47)					
					Mann–Whitney		RANCOVA^a^	
	M (SD)	Mdn	M (SD)	Mdn	U	*p*	F	*p*
The AMT								
Specific memory recall	5.21(1.94)	5	7.43(1.77)	7	492.5	< 0.001***	13.3	< 0.001***
Non-specific memory recall	3.13(1.47)	3	2.11(1.54)	2	783	0.001**	4.66	0.033^b^
Extended memory recall	2.40(1.38)	2	1.55(1.19)	1	818	0.002**	3.92	0.051
Categorical memory recall	0.45(0.61)	0	0.34(0.60)	0	1114	0.276		
Non-memory recall	0.28(0.72)	0	0.21(0.51)	0	1237	0.928		
Omission/no respond	1.66(1.51)	1	0.47(1.12)	0	581.5	< 0.001***	11.03	0.001**
Overall score^c^	29.21(6.27)	29	35.26(4.85)	36	522	< 0.001***	11.4	0.001**

*** *p* < *0.0*01, ** *p* < *0.0*1, * *p* < 0.05. ^a^ Covariates were age, alcohol use, GHQ and PSQI. ^b^ This did not survive the Holm–Bonferroni correction. ^c^ Responses were scored from 4 (specific) to 0 (omission) and summed for an overall score.

Additional analyses were conducted to further explore the effects of substance use on AM. In the first analysis, participants who had previously used substances but no longer did, as well as those who reported very rarely substance use (e.g., once or twice a year) were excluded to mitigate the potential impact of single-time substance use (see Table S2 in Supplementary Material). Subsequently, a more restrictive analysis was performed, excluding participants with both frequent (1 or 2 times a week) and very frequent substance use (3 or more times a week) in addition to the previously excluded groups. This analysis aimed to isolate the potential impact of moderate substance use on group differences, while minimising the influence of extreme usage patterns (see Table S3 in Supplementary Material). Overall, the majority of results remained unchanged.

## Discussion

The present study assessed the detailed effects of recreational substance use on AM to provide a better understanding of the specific aspects of AM that are most vulnerable to substance use. The results revealed that participants who reported recreational substance use, primarily occasional use of polysubstance, appeared to recall fewer specific personal event memories and more non-specific memories, particularly extended memories, compared to those who did not report such use. They were also less likely to respond within the allotted time. These findings are in line with the literature^33–35^. While the majority of past research has primarily focused on the impairment of autobiographical memory among individuals with frequent cannabis use or substance dependence, the current study extends this understanding by demonstrating that even sporadic substance use can also have a detrimental impact on autobiographical memory.

One of the well-accepted models for understanding autobiographical memory recall is the Self-Memory System (SMS), developed by Conway and Pleydell-Pearce^53^. This model structures autobiographical knowledge hierarchically across three levels of specificity: event-specific knowledge (ESK), general events, and lifetime periods. At the broadest level, lifetime periods encompass major phases in life (e.g., “college years”), while general events represent more specific sequences or repeated activities within those phases (e.g., “taking tests during college”). The most detailed level, ESK, involves vivid recollections of individual events (e.g., “the final exam in the last year of college”). Retrieving an autobiographical memory requires navigating this hierarchy, typically starting from a general level and progressively becoming more specific and detailed through generative and elaborative processes^54,55^. However, various factors can interfere with this process, causing the search to be prematurely aborted and resulting in more generalised rather than specific memories.

The tendency among participants who reported substance use to recall more non-specific than specific memories suggests difficulties in navigating the hierarchy. This failure to remember specific memories is also known as over-general memory which is most pronounced in clinical populations, including individuals with major depressive disorder^56,57^. Given the high prevalence of depression among participants who reported substance use^58,59^, this may partly explain the AM deficits observed in this population.

Another contributing factor to over-general memory may be reduced executive function, which limits a person’s ability to focus on retrieval and inhibit interfering thoughts (Williams et al.^57^). For instance, Guler and Mackovichova^60^ showed that people with higher levels of executive function skills, notably higher levels of cognitive flexibility and inhibitory control remembered much more specific memories than people with lower levels of executive function skills. Given the well-documented impairment of executive functions in people with a history of substance use^24,61–67^, this may explain their difficulties in accessing specific memories.

Additionally, encoding issues may play a role. Existing research indicates that substance use can impair time processing^68,69^ which might lead to poor encoding of detailed information. This could result in the encoding of information in larger, less specific chunks, reducing temporal resolution. As a consequence, individuals may struggle to retrieve precise memories over time, leading to the recall of broader, less specific memories.

Participants with a history of substance use were also less likely to respond within the given time, potentially due to general memory deficits such as difficulties with information retrieval. They may require additional time or a more effective search strategy to locate and recall relevant information. This idea is consistent with existing research suggesting that people who use substance struggle more with retrieval than with encoding^70^. For example, studies by Gouzoulis-Mayfrank et al.^71^ and Woods et al.^72^ found that individuals with a history of substance use performed worse on free recall tests (requiring them to retrieve information without prompts), compared to individuals without a history of substance use, while their performance on recognition tests, where they identified previously encountered information, showed no significant difference. This pattern suggests that while individuals with a history of substance use may encode information effectively (intact availability), they experience difficulties recalling it without cues (impaired accessibility).

Autobiographical memory serves multiple functions^32^, including directing future actions^73^ by establishing successful behaviour models from past experiences encoded with rewards and losses^74^, building social bonds^75^, and developing the personal identity and the continuity of the self^76^. AM also helps to alter undesirable moods or maintain desirable moods by recalling positive personal experiences, hence it is important for emotional resilience^77^. The deficit in emotional resilience also accounts for the lower scores on the GHQ and feed into sleep disturbances as shown on the PSQI. Consequently, impaired AM might result in deficits in these functions which, in turn, can cause personal and social problems that may lead to substance use or contribute to the maintenance of substance use and, in some cases, the transition from recreational substance use to substance dependence.

### Limitations and future directions

The limitations in this study reflect common challenges in neurocognition research involving individuals with a history of substance use. These include issues like polysubstance use, difficulties quantifying substance consumption, legal constraints on collecting such data and reliance on self-report to confirm abstinence. While it is possible that some participants may have inaccurately reported their abstinence status, raising the possibility that some observed effects could reflect acute or sub-acute rather than long-term consequences, a recent meta-analysis indicates that agreement between self-report and biological testing of substance use is generally high. This suggests that self-report remains a valid and reliable method for assessing substance use in research contexts^78^. Furthermore, it is possible that the groups may have varied in some variables beyond recreational substance use. Although variables like IQ, general health, sleep quality, and age were controlled for, others, such as nicotine use, could not be fully accounted for. Furthermore, detailed information on historical patterns of substance use among individuals who had stopped using substances, including alcohol, was not collected. As a result, potential effects on autobiographical memory performance may have gone unmeasured. Additionally, emotional cue-word tasks may not accurately reflect natural memory retrieval patterns, where memories are often recalled spontaneously. Future research should address these limitations by using more naturalistic methods, such as autobiographical memory sentence-stem tasks^79^, confirming abstinence with substance testing (e.g., saliva or urine), and evaluating lifetime substance use, potentially with tools like the Timeline Drug Use Calendar^80^.

## Conclusion

The detailed effects of recreational substance use on AM were examined in this study to provide a better understanding of the specific aspects of AM that are most vulnerable to substance use. The results revealed that individuals with a history of substance use recalled significantly fewer specific personal event memories than individuals without a history of substance use. They were also less likely to respond within the allotted time. The findings contribute to the body of research on substance-related impairments in autobiographical memory.

## Supplementary Information

Supplementary Information.

## Data Availability

The datasets generated and analysed during the current study are available in the Open Science Framework repository, [10.17605/OSF.IO/VUJ3T].
