# Supplementary material for: Recreational substance use is linked with difficulty in recalling personal experiences
Source: Sci Rep. 2025 Oct 3;15:34492. doi: 10.1038/s41598-025-13800-y (PMC12494744; doi:10.1038/s41598-025-13800-y)
Supplement: Supplementary file 1 — Supplementary Information. [file 41598_2025_13800_MOESM1_ESM.docx]

**Supplementary Material**

Illegal Recreational Drug Use is Linked with Difficulty in Recalling Personal Experiences,

Adnan Levent & Eddy Davelaar; Royal Holloway, University of London, adnan.levent@rhul.ac.uk

**Figure S1. Overview of drug use profiles with the number of participants for each profile which provides a breakdown of the polydrug usage**.


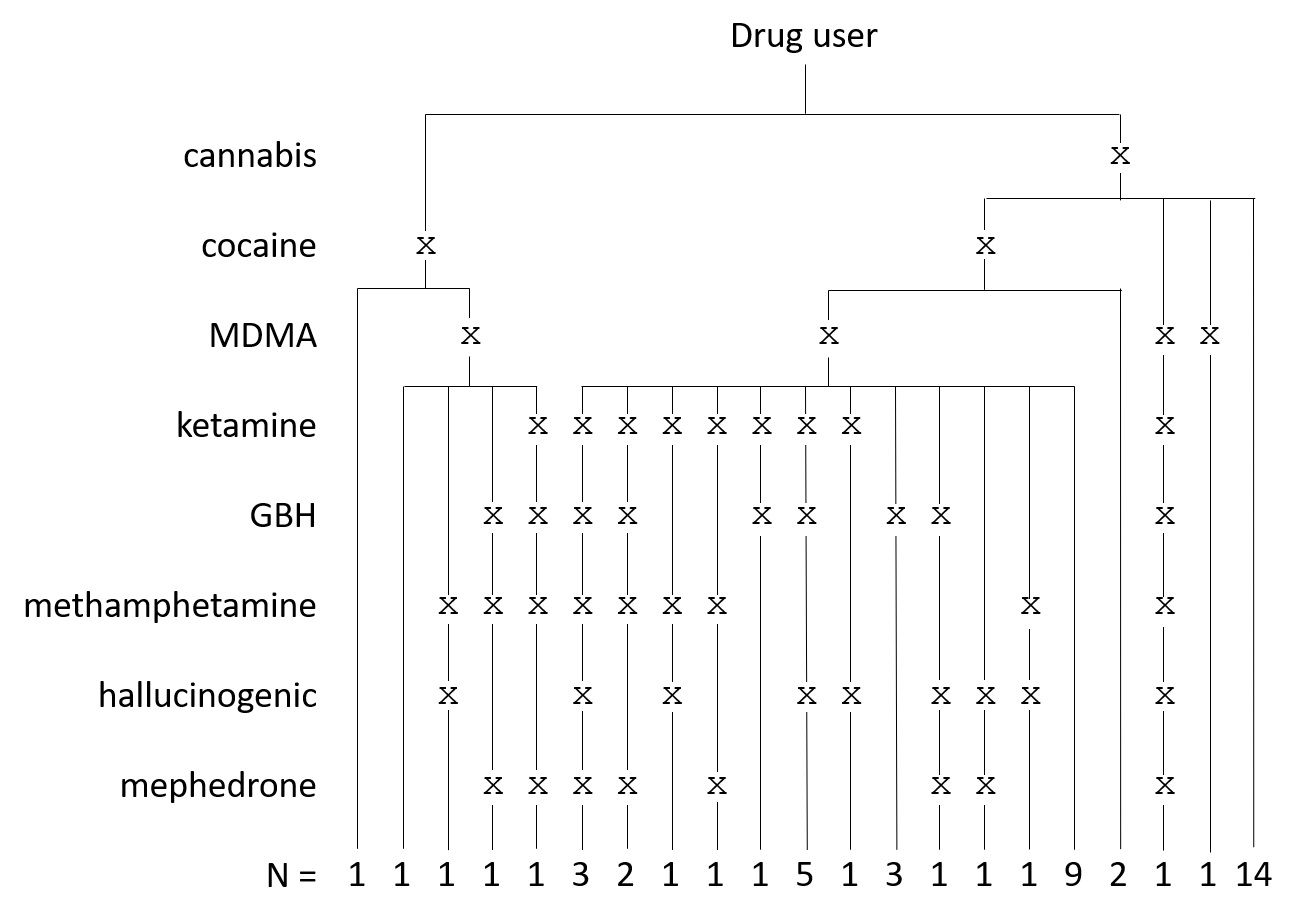


**Table S2: Mann-Whitney and RANCOVA tests’ results for the AMT after removing ex-users and those who very rarely used drugs (1 or 2 times a year) users.**

|  | **Drug User (31)** | | **Non-Users (47)** | |  | | | | | |
| --- | --- | --- | --- | --- | --- | --- | --- | --- | --- | --- |
|  |  |  |  |  | **Mann-Whitney** | | **RANCOVA** | | | |
|  | ***M (SD)*** | **Mdn** | ***M (SD)*** | **Mdn** | ***U*** | ***p*** | ***F*** | | ***p*** |  |
| **The AMT** | | | | | | | | | | |
| Specific memories recall | 4.87(1.43) | 5 | 7.43(1.77) | 7 | 204 | <.001*** | 15.28 | <.001*** | | |
| Non-specific memories recall | 3.26(1.24) | 3 | 2.11(1.54) | 2 | 414.5 | .001** | 4.92 | .030^b^ | | |
| Extended memories recall | 2.42(1.29) | 2 | 1.55(1.19) | 1 | 463.5 | .005** | 4.28 | .042^b^ | | |
| Categorical memories recall | 0.58(0.67) | 0 | 0.34(0.60) | 0 | 581 | .076 | 1.01 | .318 | | |
| Non-memories recall | 0.26(0.68) | 0 | 0.21(0.51) | 0 | 726 | .969 | .000 | .986 | | |
| Omission/no respond | 1.87(1.34) | 2 | 0.47(1.12) | 0 | 263 | <.001*** | 12.84 | <.001*** | | |
| Positive |  |  |  |  |  |  | 9.13 | .003** | | |
| Negative |  |  |  |  |  |  | 7.82 | .007** | | |
| Overall score | 28.16(5.28) | 28 | 35.26(4.85) | 36 | 216 | <.001*** | 12.58 | <.001*** | | |

*** *p* < .001, ** *p* < .01, * *p* < .05. a Covariates were age, alcohol use, GHQ and PSQI. ^b^ This did not survive the Holm-Bonferroni correction.

**Table S3: Mann-Whitney and RANCOVA tests’ results for the AMT after removing frequent (1 or 2 times a week) and very frequent drug users (3 or more times a week), as well as ex-users and very rarely drug users.**

|  | **Drug User (23)** | | **Non-Users (47)** | |  | | | | |
| --- | --- | --- | --- | --- | --- | --- | --- | --- | --- |
|  |  |  |  |  | **Mann-Whitney** | |  | | |
|  | **M (SD)** | **Mdn** | **M (SD)** | **Mdn** | **U** | **p** |  |  |  |
| **The AMT** | | | | | | | | | |
| Specific memories recall | 5(1.45) | 5 | 7.43(1.77) | 7 | 164 | <.001*** |  |  |  |
| Non-specific memories recall | 3.26(1.29) | 3 | 2.11(1.54) | 2 | 309.5 | .003** |  |  |  |
| Extended memories recall | 2.35(1.20) | 2 | 1.55(1.19) | 1 | 348 | .013* |  |  |  |
| Categorical memories recall | 0.65(0.71) | 1 | 0.34(0.60) | 0 | 406 | .048* |  |  |  |
| Non-memories recall | 0.26(0.69) | 0 | 0.21(0.51) | 0 | 537.5 | .954 |  |  |  |
| Omission/no respond | 1.74(1.32) | 2 | 0.47(1.12) | 0 | 207.5 | <.001*** |  |  |  |
| Overall score | 28.61(5.17) | 28 | 35.26(4.85) | 36 | 171 | <.001*** |  |  |  |

*** *p < .*001, ** *p < .*01, * *p* < .05..
